# Corporis Humani Fabrica of Andreas Vesalius

**Published:** 1915-03

**Authors:** 


### Corporis Humani Fabrica of Andreas Vesalius.

These two volumes on the human anatomy by Andreas Vesalius, physician to Charles V, were given me by Rev. Father Amadeus Guyol, at one time in charge of the parochial school at Twelfth and Avenue I, Galveston, Texas, as a Christmas present, and though they were a family heirloom, he did not know how long they had existed in his family, but, owing to their historical importance and the fact that I had once been a teacher of anatomy and having resigned voluntarily from teaching, and refusing the chair of anatomy in the Medical Department of the University of Texas when it was first organized, but having always taken a great interest in the study of anatomy, I concluded I could do nothing better than to present these rare works to the anatomical branch of the Medical Department of the University of Texas.

The following article, including the introductory paragraphs of an editorial in the October, 1914, number of the *Interstate Medical Journal,* I respectfully attach, because it is a concise history of Andreas Vesalius, showing that he was the first real anatomist who learned his anatomy in toto from the human being and not from monkeys and other two-legged animals.

The mere fact that Louvain was recently destroyed, the place from which Andreas Vesalius received his first training as a medical man, gives added interest to these historic volumes.

A. W. FLY, M. D.,
Board of Regents, University of Texas.

Below are the interesting paragraphs introducing the editorial:

"It would be meet at this time to enter into a discussion as to the real reasons why so famous a university as that of Louvain was destroyed in the present war, but all scholars must bemoan the fact that this calamity, this blow to culture, has occurred. What student of the history of medicine has not lovingly referred, time and again, to the venerable pile of stone which typifies the University of Louvain or has not visited it for the sake of the reputation of one man—Andreas Vesalius. Let us hope that in the future when the jars and wranglings that are besetting the world at present are less virulent, when the sanity of outlook again asserts itself, when men shall write with their mind's content undisturbed, the regret at the destruction of this university will only be that of the inevitable loss of a dear friend. In the meanwhile let us read and re-read that striking essay on Vesalius, 'Anatomy in Long Clothes,' from the pen of Henry Morley, himself a physician and a writer of no mean parts, which first saw the light of day in *Fraser's Magazine,* November, 1853. And it is in the hope that the classical prose of this now almost forgotten essay will revive an interest in Vesalius and bring to the notice of the medical men of this country the great loss of a historic building, that the following lines are quoted:

" 'There is an old folio, known to most men who have visited the fountain-heads of medical literature, and dear to bookworms for its wood cut illustrations, which in their own time were ascribed to Titian. It is the Corporis Humani Fabrica of Andreas Vesalius. The first page is adorned with a large and spirited wood cut, in which a young man, wearing professor's robes, is to be seen standing at the table of a lecture theater, and pointing out from a robust subject that lies before him the inner secrets of the human body. The tiers of benches that surround the lecture table are completely crowded with grave doctors, who are leaning forward, struggling to see, and even climbing upon railings, from which they look down with faces that present a striking group, expressive of much wonder, interest and curiosity, mixed with a little awe. And yet they look upon a spectacle which is presented in our day as a matter of course to thousands of young men during the winter session at the hospitals.

" 'The wood cut at once leads us to suppose that we have to deal in the book to which it is prefixed with a man who was the first to force his way into a path obstructed by a heavy barricade of prejudice. If we turn over a leaf we find his portrait in another sketch, rough, bold, and masterly. It portrays spirit and flesh of a young man who has the marks of a hard working brain upon his forehead and of a firm will upon his face. He looks like a man born to do work for the world, and not unwilling at the same time to take ease in it. He evidently can enjoy as well as think, and will, and do. His beard is very trim, his senses look acute, his rather handsome features express much refinement, aptness also for a look of scorn. He shows like a chief in intellect, a gracious king over some region of knowledge, who possesses all he could inherit, and knows how to conquer more; a good companion to kindred minds when recognized among them as a leader. So we judge from the noble portrait of the young professor in his robes, Andreas Vesalius, aged, as we are told by the inscription on the border, twenty-eight; a man who at that age had already become the Luther of Anatomy.' "

---

WANTED.—To buy now, second-hand microscope, THREE lenses; MUST be good, including oil emersion lens. Address R. H. Crockett, M. D., Roundrock, Texas.

---

HER IDEA OF IT.—"Yes," said the good woman who was describing the last illness of a friend, "she was taken suddenly sick with pantomine poisoning, and four doctors came to the house and insulted about her and diagrammed her case very closely. They decided that she had eaten some fish or something that had paragraphs in it, and so they gave her a hypercritical injection of a serial that would destroy the basilica, but she didn't seem to help any, and she soon was in a state of chromo."—*Exchange.*

